# Evaluation of adherence to guideline-directed therapy and risk factors for exacerbation in mild asthma: a retrospective chart review

**DOI:** 10.1186/s13223-024-00888-6

**Published:** 2024-03-28

**Authors:** Beth A. Zerr, Jacklyn M. Kruse, Jon J. Glover

**Affiliations:** 1 University of Arizona, 85724 Tucson, AZ PO 243002, USA; 2 Community Health Network, 3508 S Lafountain, 46902 Kokomo, IN USA; 3 Outcomes and Analytics, Pfizer Medical, Gilbert, AZ USA

**Keywords:** Asthma, Inhaled corticosteroids, Mild asthma, Asthma exacerbations, Exacerbation, Corticosteroids, SABA, Reliever medication, Mild persistent asthma, GINA

## Abstract

**Background:**

A significant update was made to both the Global Initiative for Asthma (GINA) in 2019 and the National Heart Lung and Blood Institute (NHLBI) asthma guidelines in 2020 for mild asthma. These groups no longer recommend short-acting beta-agonists (SABA) as monotherapy for mild (GINA) or mild-persistent (NHLBI) asthma. With the lag that can occur between guideline or evidence updates and changes in practice, this study sought to evaluate whether guideline adoption had occurred.

**Methods:**

In this retrospective chart review, patient electronic medical records from a large healthcare system were evaluated from July 1 of 2021 to July 1 of 2022 to determine how many patients with mild asthma were prescribed as needed or daily inhaled corticosteroids (ICS) in addition to as needed SABA. The secondary outcome was to evaluate the incidence of exacerbations in patients with mild asthma, comparing those on guideline-directed therapy or not. In addition, we evaluated other patient factors increasing exacerbation risk in mild asthma.

**Results:**

For the primary outcome, of the 1,107 patients meeting inclusion criteria, 284 patients (26%) did not have documentation of guideline-directed therapy for mild asthma during the study period, while 823 (74%) were on guideline-directed therapy (Diff:48.7%; 95% CI:45.1 to 52.3%, *p* < 0.001). For the secondary objective, 161 patients had an exacerbation (12% on guideline-directed therapy, 15.4% not on guideline-directed therapy). This difference in incidence of exacerbation between the two treatment groups was not statistically significant (Diff: -3.4%; 95% CI: -8 to 1.1%; *p* = 0.133). In addition, being female, having GERD, and being obese were all statistically significant factors associated with having asthma exacerbations among our patient population.

**Conclusions:**

Nearly one-fourth of patients with mild persistent asthma were not on guideline-directed therapy, despite updates in asthma guidelines (GINA 2019, NHLBI 2020). Factors such as being female, having GERD, and being obese were all statistically significant factors associated with having asthma exacerbations among patients with mild persistent asthma.

## Background

Asthma is a chronic respiratory disease characterized by airway inflammation, bronchial hyperresponsiveness, and reversible airflow obstruction [1]. Asthma is commonly classified as mild, moderate, or severe based on symptom frequency and severity, and these classifications guide treatment with the goals to reduce symptoms and prevent asthma exacerbations. The term mild asthma can be misleading, implying a low risk of serious disease sequelae. Because of this, some organizations have proposed no longer using the term mild to describe asthma [2]. According to the Centers for Disease Control (CDC), 25 million people in the United States are currently diagnosed with asthma, and a majority (50–75%) classified as having mild asthma [3, 4]. Of those diagnosed with asthma, over 41% claimed to have one or more asthma exacerbations in a 12-month period, while nearly 2 million visited the emergency room due to uncontrolled asthma [3]. In addition, per a systematic review published in 2020, up to 22% of patients with mild asthma had a severe exacerbation in the previous year [5]. An earlier study published in 2007 found severe exacerbations in mild asthma represent 30–40% of asthma exacerbations requiring emergency consultation [6], highlighting the need for more effective treatment of mild asthma.

The Global Initiative for Asthma (GINA) and the National Heart Lung and Blood Institute (NHLBI) updated their asthma guidelines in 2019 and 2020, respectively. Both groups included a significant update to the recommended treatment of mild asthma, and the recommendation remains in the latest guideline updates. Before the 2019 GINA update, short-acting beta-agonist (SABA) monotherapy was considered appropriate only for step 1 therapy (mild intermittent asthma or very mild asthma). The fundamental change in the 2019 GINA guideline was the recognition that SABA monotherapy was no longer appropriate for any patient with asthma, regardless of severity classification. The 2020 NHLBI update continued to recommend SABA monotherapy for mild intermittent asthma, but no longer recommended it for mild persistent. Specifically, the use of daily inhaled corticosteroids (ICS) + as needed SABA, as needed ICS-long-acting beta agonist (LABA) or as needed ICS + as needed SABA are recommended over monotherapy with as needed SABA [2, 7]. This change was prompted by evidence of ICS-containing treatment markedly reducing asthma hospitalizations, severe exacerbations, and death [8–10]. As these updates represent fundamental changes to the management of mild asthma, changes in prescribing patterns may lag behind guideline publication.

The purpose of this study was to evaluate how many patients with mild asthma were prescribed as needed or daily ICS in addition to as needed bronchodilator per the updated GINA and NHLBI guidelines. In addition, evaluation of incidence of exacerbations in patients with mild asthma, and examination of patient-specific factors that contribute to exacerbations, included but not limited to guideline-adherence, will also be assessed.

## Methods

In this retrospective chart review, patient electronic medical records were evaluated from Banner Health, a large healthcare system with 30 hospitals and over 300 clinics across six states in the western part of the United States. This study was approved by Banner Health IRB. Charts were reviewed to determine how many patients with mild asthma were prescribed as needed or daily ICS in addition to as needed SABA. Charts were electronically reviewed from July 1, 2021, to July 1, 2022. Patients were included if they were a primary care patient age 12 years or older with an ICD-10 code(s) for mild or mild persistent asthma {ICD-10 codes: J45.30 (mild persistent asthma, uncomplicated), J45.31 (mild persistent asthma with (acute) exacerbation), J45.32 (Mild persistent asthma with status asthmaticus)}. A patient with a prescription, lab work, and/or encounter generated during study duration was considered an active patient. Patients were excluded if they were less than 12 years of age, had an active diagnosis for moderate persistent asthma or severe persistent asthma, an allergy to ICS-containing medications, or an active prescription for a nebulizer solution. The following data were collected: age, sex, race, payer type, active prescriptions for ICS, SABA, and ICS-LABA medications, prescriber, encounter diagnoses codes, location name, and date. The primary outcome was to evaluate how many patients with mild asthma were prescribed as needed or daily ICS in addition to as needed SABA. The secondary outcome was to evaluate the incidence of exacerbations {ICD-10 code: J45.901(unspecified asthma with (acute) exacerbation)} in patients with mild asthma, comparing those on guideline-directed therapy (daily ICS + as needed SABA, as needed ICS-LABA or as needed ICS + as needed SABA) or not (as needed SABA only). In addition to guideline-adherence, we evaluated other patient factors influencing exacerbation risk in mild asthma.

### Statistical and data analysis

Categorical variables were reported as counts and percentages with differences between groups using chi-square, two-proportion, or Fisher’s exact test. Continuous measures were reported with means and standard deviation and/or, medians with interquartile ranges and differences between groups using student t-test. Lastly, a multivariate logistical regression was used to evaluate demographic and clinical characteristics most influencing asthma exacerbations. Minitab v20 was used for all statistical comparisons with alpha = 0.05.

## Results

**Fig. 1:**
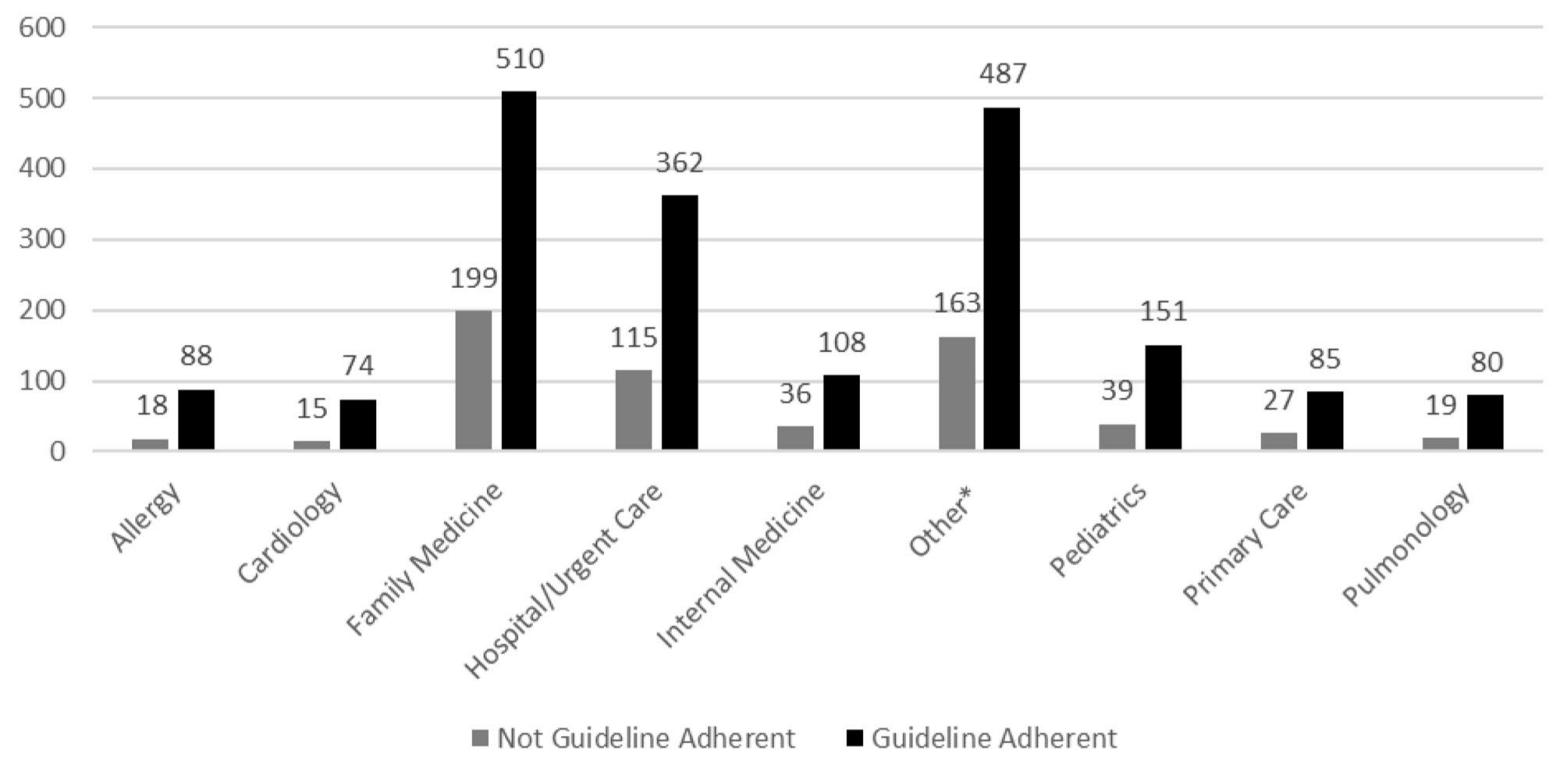
Facility/provider type and guideline adherence *Includes other specialty clinics with a small representation such as Obstetrics/Gynecology., Ophthalmology, Neurology, Urology, Dermatology, Gastrointestinal, etc Looking at this graph we see provider type and whether or not the patient was on guideline therapy. It may be hard to attribute patients to just one facility because they could be going to 2–3 different providers listed here. The numbers here add up to over 2,500, which is well above our 1,107 patients in our inclusion group

Between July 1, 2021 and July 1, 2022, there were 1,107 patients who met inclusion criteria for this study. In evaluating the primary outcome, of those 1,107 patients, 284 patients (26%) did not have documentation of guideline-directed therapy for mild asthma during the study period, while 823 (74%) were on guideline-directed therapy (Diff:48.7%; 95% CI:45.1 to 52.3%, *p* < 0.001). The mean age of those included in the study was 42.4 years for non-guideline-adherent patients and 43.7 years for guideline-adherent patients (*p* = 0.353) (Table 1). In addition, most of the population was female and Caucasian/white, with no statistical significance between the primary outcome groups (*p* = 0.781 and *p* = 0.524, respectively) (Table 1). When examining provider type in respect to the primary objective (non-guideline-adherent versus guideline-adherent), 37% of patients seen in the primary care setting (primary care, family medicine and internal medicine clinics) have no documentation of guideline-directed therapy (Fig. 1).

**Table 1:** Demographics

Characteristic	Non-Guideline-Adherent (*n* = 284)	Guideline-Adherent (*n* = 823)	Diff	95% CI	***P*** value
**Age in years**					
Mean (SD) Median (IQR)	42.4 (20.7) 42 (37)	43.7 (21.5) 42 (39)	-1.3	-4.2 to 1.5	0.353
**Sex (%)**					
Female Male	173 (60.9) 111 (39.1)	509 (61.9) 314 (38.1)	-1 1	-7.5 to 5.6 -5.6 to 7.5	0.781
**Race (%)**					
Caucasian/White Other*	216 (76.9) 68 (23.1)	631 (77.0) 192 (23.0)	-0.1 0.1	- -	0.524

*Other groups: American Indian, Asian/Pacific Islander, Black/African American, Hispanic, Middle Eastern Indian, Native Hawaiian or Other Pacific Islands, two or more races, refused or unknown

When assessing the secondary objective, 161 patients of the 1107 had an exacerbation during the study period. Of patients not on guideline-directed therapy, 12% experienced an exacerbation. Of patients on guideline-directed therapy, 15.4% had an exacerbation. This difference in incidence of exacerbation between the two treatment groups was not statistically significant (Diff: -3.4%; 95% CI: -8 to 1.1%; *p* = 0.133).

**Fig. 2:**
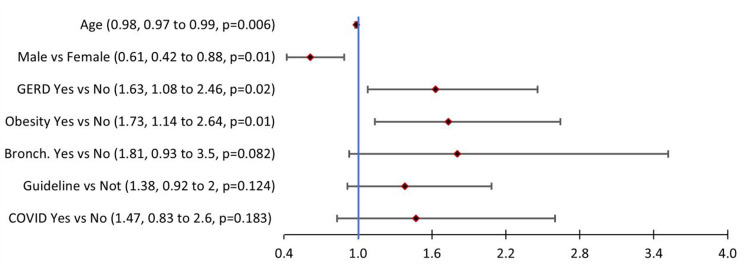
Logistical regression model (*n* = 1107): Factors associated with at least one asthma exacerbation* *Multivariate logistical regression including factors creating most parsimonious model. Overall model *p* < 0.001, Concordance = 63.8%, R^2^ = 3.57%, Hosmer-Lemeshow *p* = 0.741

The multivariate logistical regression model (see Fig. 2) represents those demographic and clinical characteristics most influencing an asthma exacerbation (*p* < 0.001). As shown in the figure, asthma exacerbations decreased incrementally by 1.2% for each additional year of age (*p* = 0.006). Males were about 39% less likely to have an exacerbation when compared to females (*p* = 0.01). Those with gastroesophageal reflux disease (GERD) were 63% more likely to have an asthma exacerbation (*p* = 0.02). Obese patients were 73% more likely to have an asthma exacerbation (*p* = 0.01), while those with bronchitis being about 81% more likely to have an asthma exacerbation, but this was not statistically different (*p* = 0.082). Lastly, three characteristics trended towards increased incidence of exacerbation, but were not statistically significant; patients with documented guideline-directed therapy were 38% more likely to have an asthma exacerbation, with documented history of Covid were 47% more likely, and with bronchitis were 81% more likely (*p* = 0.124, *p* = 0.183, and *p* = 0.082 respectively). In conclusion, being female, having GERD, and being obese were all statistically significant factors associated with having asthma exacerbations among our patient population. Other factors that were assessed but did not fit the logistical regression model were COPD, pneumonia, influenza, allergic rhinitis or sinusitis, smoking, and heart failure.

## Discussion

The results of this study indicate that about 3 in 4 (74%) patients with mild asthma did have documentation of guideline-directed therapy. Previously reported adherence rates to asthma guidelines have varied. A 2016 study assessed primary care adherence to the previous 2007 NHLBI asthma guidelines. This study found that 88% of patients had documented guideline adherence for reliever medication and 70.4% had guideline adherence to maintenance medication [11]. Data on adherence to the more recent guidelines is available from an international study that assessed adherence via provider and patient surveys. This 2021 study examined asthma therapy in four countries (Australia, Canada, China, and the Philippines) and found that 47% of patients were on guideline-directed therapy [12]. Our study found a greater percentage of patients on guideline-directed therapy (74% compared to 47%). This may be due to the increased amount of time from the updated guidelines release and/or data collection methodology.

Information in Fig. 1 allows an assessment of which facility had the greatest patient population not receiving guideline-directed therapy, which may help target providers with education on guideline updates. As shown in the results and in Fig. 1, patients seen in the primary care setting (primary care, family medicine and internal medicine clinics) had the highest percentage of patients without guideline-directed therapy. This is valuable information as most patients with mild asthma will often be seen in a primary care setting due to the low severity of their asthma symptoms.

Regarding the secondary objective, patients in this study were found to be at a slightly greater risk for an asthma exacerbation if they were on guideline-directed therapy versus not; however, this objective was not statistically significant. The correlation of the timing of the asthma exacerbation and when the patient was started on guideline-directed therapy was unable to be determined based on the data gathered. During the study period, we assessed if the patient had an asthma exacerbation and if they were on guideline-directed therapy, but we did not assess the time at which these events occurred or in what order they occurred. In other words, during the study period, a patient who was not on guideline-directed therapy may have experienced an asthma exacerbation, and then was subsequently started on guideline-directed therapy. In addition, more acute or critical patients who were at a higher risk of having an asthma exacerbation may have been followed more closely by their practitioner which is why they were started on guideline-directed therapy sooner than other patients. Despite being followed more closely, they still had an asthma exacerbation due to being a higher risk patient. Finally, it is possible that some patients classified as having mild asthma actually have a more severe form of asthma and this may have contributed to the incidence of exacerbations.

Factors that were statistically significant with regards to exacerbation risk include female sex, GERD, and obesity. Previous studies have described morbidity and mortality risk factors for asthma, including high SABA use, increased age, ever smoking, and high blood eosinophils [13]. Few studies have specifically examined risk factors in patients characterized as having mild asthma. As discussed earlier, patients with mild asthma make up a large proportion of all patients with asthma, and these patients still experience exacerbations, but may not be treated with guideline-directed ICS therapy which is proven to reduce exacerbation risk [14]. Our study may add new insight into risk factors and treatment goals for patients with mild asthma, particularly optimizing treatment for GERD and obesity.

### Limitations

Despite Banner Health having a large patient population, the number of patients meeting our inclusion criteria was low. This may be suggesting a low prevalence of mild persistent asthma, low documentation of patients as having mild asthma, or more severe patients because of the large influence of the academic facilities on excluded patients. The lower than expected number of patients in this study may have limited the results.

Additionally, a limitation to the study was our inability to correlate the timing of patient exacerbations and medication use. As a result, the number of patients on guideline-directed therapy who experienced an asthma exacerbation may have been falsely elevated.

Another limitation to this study was that it was difficult to determine which provider or facility was managing therapy because patients could have been visiting multiple facilities within the institution or even outside the institution. Lastly, we are unable to be certain that each patient had the correct diagnosis code entered on their problem list. This was an assumption that the provider diagnosed the patient correctly and updated their problem list accordingly.

Accurately assessing patients with mild asthma may be a limitation of this study, as patients with more severe disease may have been classified as having mild asthma due to underreporting of symptoms by patients or failure to recognize severity by providers, particularly primary care providers.

Finally, the number of asthma exacerbations experienced by each patient is unknown based on the data gathered. This study only characterized patients as having an exacerbation or not having an exacerbation during the study period. In addition to this, patients seen at an outside facility for asthma exacerbation treatment would not be accounted for in our electronic health record. Patient adherence to medication is something we cannot determine from this study but could have an impact on exacerbations.

## Conclusion

Nearly one-fourth of patients with mild asthma in this study population were not on guideline-directed therapy, despite updates in asthma guidelines (GINA 2019 and NHLBI 2020). Factors such as being female, having GERD, and being obese are all statistically significant factors associated with having asthma exacerbations among patients with mild asthma. More work needs to be done to increase provider awareness regarding asthma guideline updates in outpatient and inpatient settings. Lastly, further studies in patients with mild asthma are needed to examine medication adherence, patient satisfaction, and exacerbation rate comparing patients on guideline-directed therapy versus those who are not.

## Data Availability

The datasets used and/or analyzed during the current study are available from the corresponding author on reasonable request.

## Abbreviations

CDC: Centers for Disease Control
GINA: Global Initiative for Asthma
NHLBI: National Heart Lung and Blood Institute
SABA: Short-Acting Beta-Agonists
ICS: Inhaled Corticosteroids
LABA: Long-Acting Beta-Agonist
